# Acquired Ventricular Septal Defect in Panton-Valentine Leukocidin-Positive Staphylococcus aureus Infective Endocarditis

**DOI:** 10.7759/cureus.44559

**Published:** 2023-09-02

**Authors:** Keng Han Yeap, David Garner, Lydia Sturridge

**Affiliations:** 1 Cardiology, London North West University Healthcare National Health Service (NHS) Trust, London, GBR; 2 Infectious Disease, Frimley Health National Health Service (NHS) Foundation Trust, Camberley, GBR; 3 Cardiology, Frimley Health National Health Service (NHS) Foundation Trust, Camberley, GBR

**Keywords:** pvl gene, pvl, panton-valentine leucocidin, staphylococcus aureus, methicillin-sensitive staphylococcus aureus, acquired vsd, acquired ventricular septal defect, vsd, ventricular septal defect

## Abstract

Infective endocarditis (IE) is life-threatening and can lead to complications if left untreated. A 56-year-old gentleman presented with acute delirium, fever and rigor. Panton-Valentine leukocidin (PVL)-positive *Staphylococcus aureus* (*S. aureus*) was isolated in the blood culture and the PR interval was prolonged on the electrocardiogram (ECG). However, the transthoracic echocardiogram (TTE) and transoesophageal echocardiogram (TOE) at presentation were unremarkable with no evidence of intracardiac vegetations. Despite expedient intravenous antibiotics, an acquired ventricular septal defect (VSD) developed, which required urgent cardiothoracic surgical repair. It is imperative to consider early surgical interventions and the use of anti-toxin antibiotics in PVL-positive *S. aureus* IE.

## Introduction

Infective endocarditis (IE) is characterised by infection of the endocardium, typically on the valvular surfaces, that has the potential to embolise distally to affect other organ systems [1]. A person is prone to developing IE when the micro-environment of the cardiac endothelium favours the adherence of microorganisms in the bloodstream, allowing them to proliferate and form infective vegetation [1]. Factors that increase one’s risk of IE include underlying structural heart anomalies, implantable cardiac devices, prosthetic heart valves, intravenous (IV) drug use, rheumatic heart diseases and immunodeficiency [1,2]. IE is life-threatening and despite the use of up-to-date and contemporaneous treatment strategies, IE still carries an inpatient mortality rate of about 15% to 20% [2]. 

IE is typically caused by bacterial pathogens, although fungal IE may rarely occur [1]. The commonest pathogens implicated in IE are *Streptococcus* spp. and *Staphylococcus* spp., whilst *Coxiella burnetii* and *Bartonella* spp. are other important causative pathogens of IE [1]. Notably, *Staphylococcus aureus* (*S. aureus)*, being a skin commensal flora, is frequently isolated in the bloodstream of IV drug users and accounts for approximately 75% of IE amongst them, with the tricuspid valve being the most commonly affected valve [3].

IE can result in various complications including septic emboli of the lungs, intracerebral abscess and splenic or digital infarction secondary to septic emboli, as well as septic embolisation to the brain which is commonly observed in *S. aureus* IE [1,3]. Another rare complication of IE is acquired ventricular septal defect (VSD) which has been occasionally reported since its first description in 1878 [4,5]. This article describes a case of acquired VSD secondary to IE caused by *S. aureus* that produces Panton-Valentine leukocidin (PVL) which is known to cause tissue necrosis [6].

## Case presentation

A 56-year-old gentleman presented to the emergency department (ED) with acute confusion, agitation, incoherent speech, fever and rigor. His past medical history of hypertension, hypercholesterolaemia and type 2 diabetes mellitus was managed with ramipril, atorvastatin and metformin, respectively. He was otherwise fit with a Rockwood Frailty Score of one, in full-time employment, consuming 50 to 60 units of alcohol weekly and without any history of IV drug use or soft tissue injury.

He was tremulous, clammy and disoriented to time, place and person. Initial vital signs showed temperature of 39°C, blood pressure of 107/64 mmHg, heart rate of 140 beats per minute (bpm), respiratory rate of 22 breaths per minute and oxygen saturation of 98% on room air. Cardiovascular, respiratory and gastrointestinal examinations were unremarkable with a clear lung field, non-elevated jugular venous pressure and no murmur. A neurological examination revealed right-sided hemineglect. His oral hygiene was good.

The electrocardiogram (ECG) revealed sinus tachycardia (149 bpm) with a PR interval of 56 ms, clear lung fields on chest X-ray and no acute intracranial pathology on brain computed tomography (CT). Admission blood tests showed acute kidney injury, a low lymphocyte count, elevated C-reactive protein, erythrocyte sedimentation rate, ferritin, serum lactate and glucose (Table 1).

**Table 1:** Blood test results on admission. Readings outside the reference range are displayed in bold.

Blood test	Result	Reference range
Hemoglobin	166 g/L	130–180 g/L
White blood cell count	8.2 × 10^9^/L	4–11 × 10^9^/L
Platelet count	159 × 10^9^/L	150–450 × 10^9^/L
Neutrophil count	7.3 × 10^9^/L	2–7.5 × 10^9^/L
Lymphocyte count	0.4 × 10^9^/L	1–4 × 10^9^/L
Erythrocyte sedimentation rate	55 mm/hour	0–28 mm/hour
Sodium	135 mmol/L	133–146 mmol/L
Potassium	4.1 mmol/L	3.5–5.3 mmol/L
Urea	12.5 mmol/L	2.5–7.8 mmol/L
Creatinine	116 mmol/L	64–104 mmol/L
Estimated glomerular filtration rate	60 mL/min	90–120 mL/min
C-reactive protein	332 mg/L	0–9.9 mg/L
Albumin	38 g/L	35–50 g/L
Ferritin	1.635 mg/L	30–300 mg/L
pH	7.33	7.32–7.43
Lactate	6.7 mmol/L	0.5–2.2 mmol/L
Glucose	20.9 mmol/L	4–11.1 mmol/L

Initial management

The patient was initially treated with ceftriaxone and aciclovir for meningoencephalitis, chlordiazepoxide for alcohol withdrawal and aspirin for left middle cerebral artery infarction. His serum lactate increased to 7.9 mmol/L despite fluid resuscitation at the ED and he required admission to the intensive care unit (ICU).

Case progression

**Figure 1:**
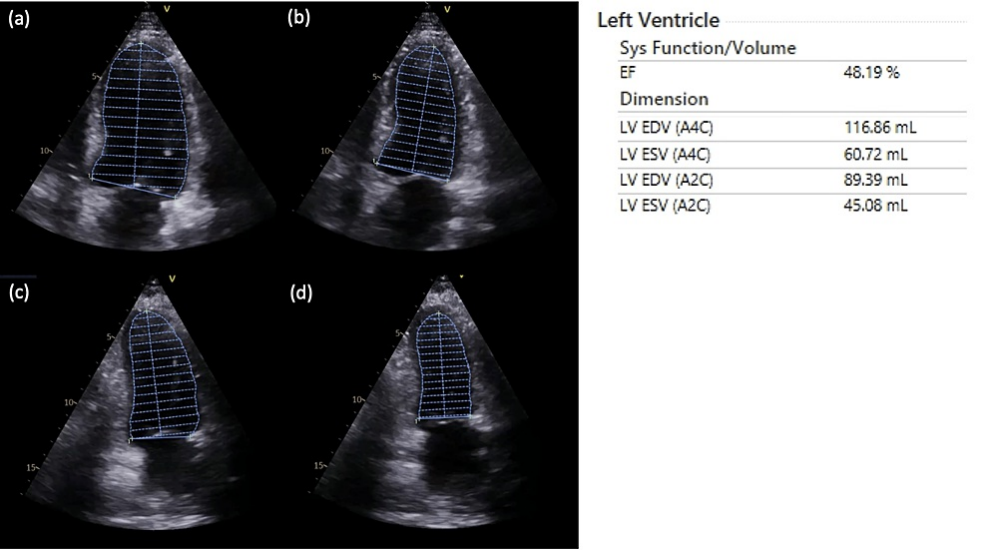
Quantification of left ventricular (LV) dimensions and ejection fraction (EF) with transthoracic echocardiography. (a) LV end-diastolic volume (EDV) in apical four-chamber (A4C) view; (b) LV end-systolic volume (ESV) in A4C view; (c) LV EDV in apical two-chamber (A2C) view; (d) LV ESV in A2C view.

Four sets of blood culture were positive for methicillin-sensitive *Staphylococcus aureus* (MSSA) with positive polymerase chain reaction for the gene encoding PVL. However, transthoracic echocardiogram (TTE) revealed left ventricular ejection fraction (LVEF) of 40% to 50% (Figure 1) and no vegetations.

**Figure 2:**
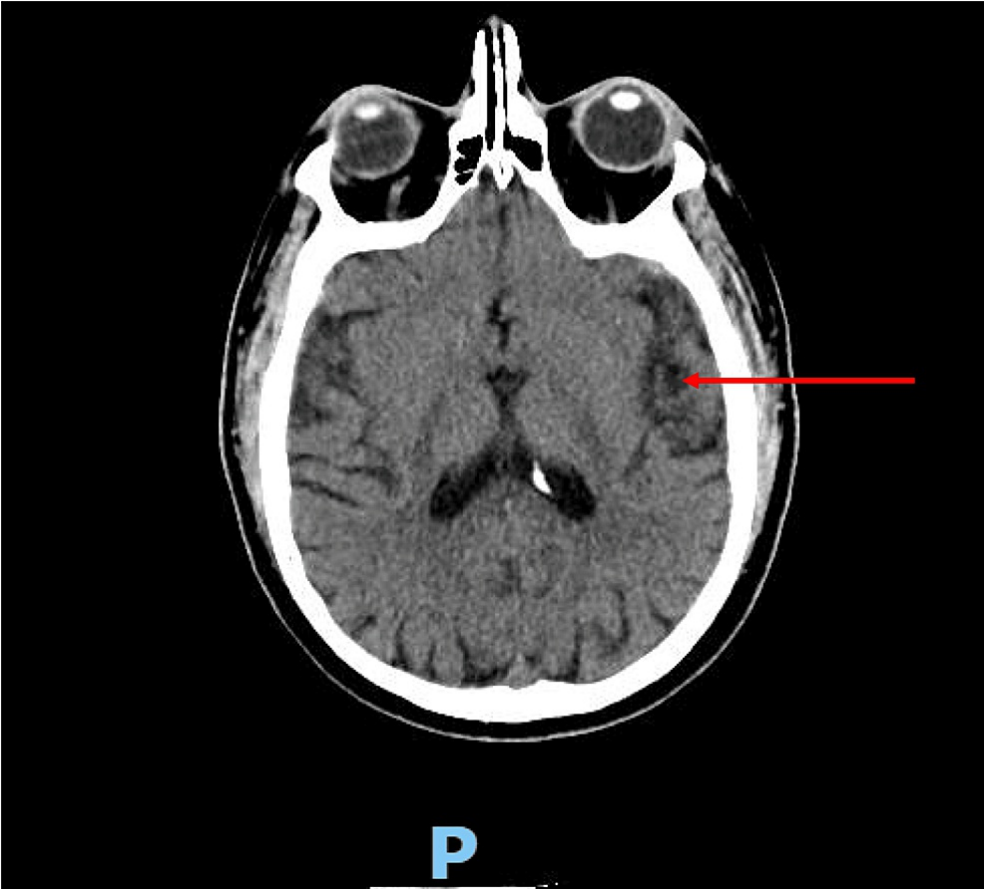
Brain computed tomography showing a focal low attenuation area representing septic emboli or meningoencephalitis.

**Figure 3:**
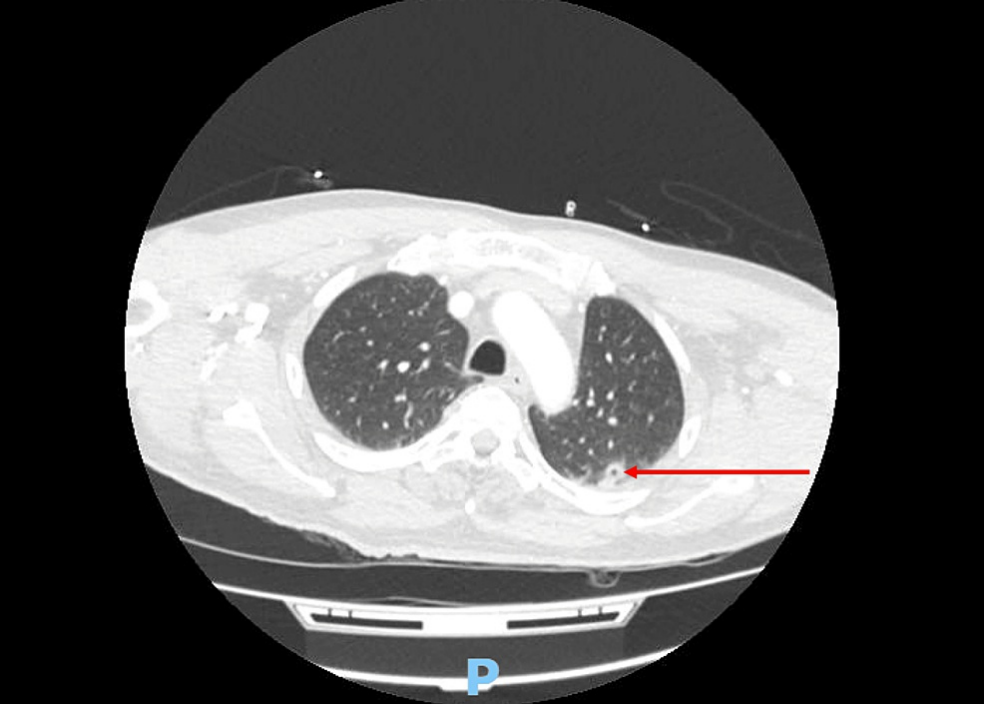
Chest computed tomography showing a cavitating nodule in the left lung apex.

**Figure 4:**
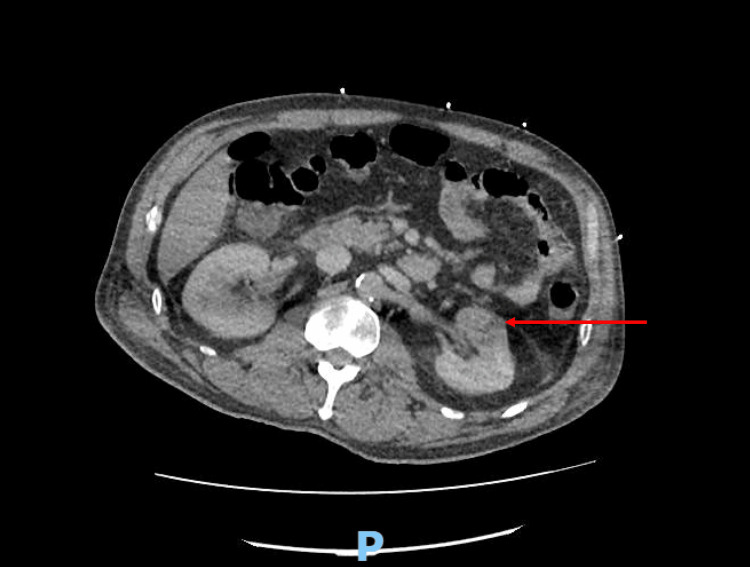
Abdominal computed tomography showing a low attenuation area in the left kidney indicating renal infarction.

A repeat brain CT demonstrated a focal low attenuation area representing septic emboli or meningoencephalitis (Figure 2). Chest CT revealed a cavitating nodule in the left lung apex (Figure 3) and abdominal CT revealed left renal infarction (Figure 4). Magnetic resonance imaging of the spine did not reveal any feature of infective discitis or cord infarction. Lumbar puncture was performed with clear cerebrospinal fluid sampled in which biochemical analysis, microscopic examination and culture results were unremarkable.

Clinical suspicion of IE

The patient was subsequently treated for PVL-positive staphylococcal IE complicated by septic embolisation with flucloxacillin, linezolid and a short course of rifampicin and gentamicin in order to control ongoing bacteraemia. He developed generalised oedema and was treated with IV furosemide. 

**Figure 5:**
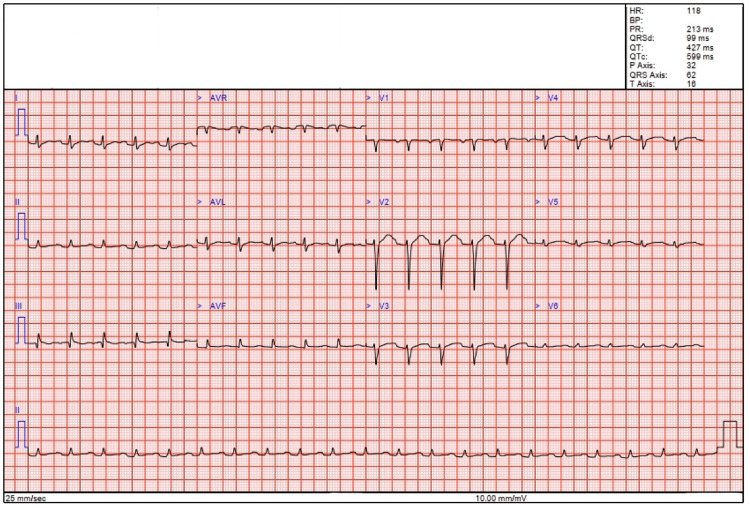
Electrocardiogram showing prolongation of the PR interval.

Four days after initial presentation and whilst still in the ICU, the PR-interval on the ECG increased to 213 milliseconds (Figure 5), but the transoesophageal echocardiogram (TOE) revealed no evidence of vegetation or para-aortic root abscess. A subsequent CT-gated contrast thoracic aorta scan showed no evidence of aortitis and ongoing medical management was recommended.

**Figure 6:**
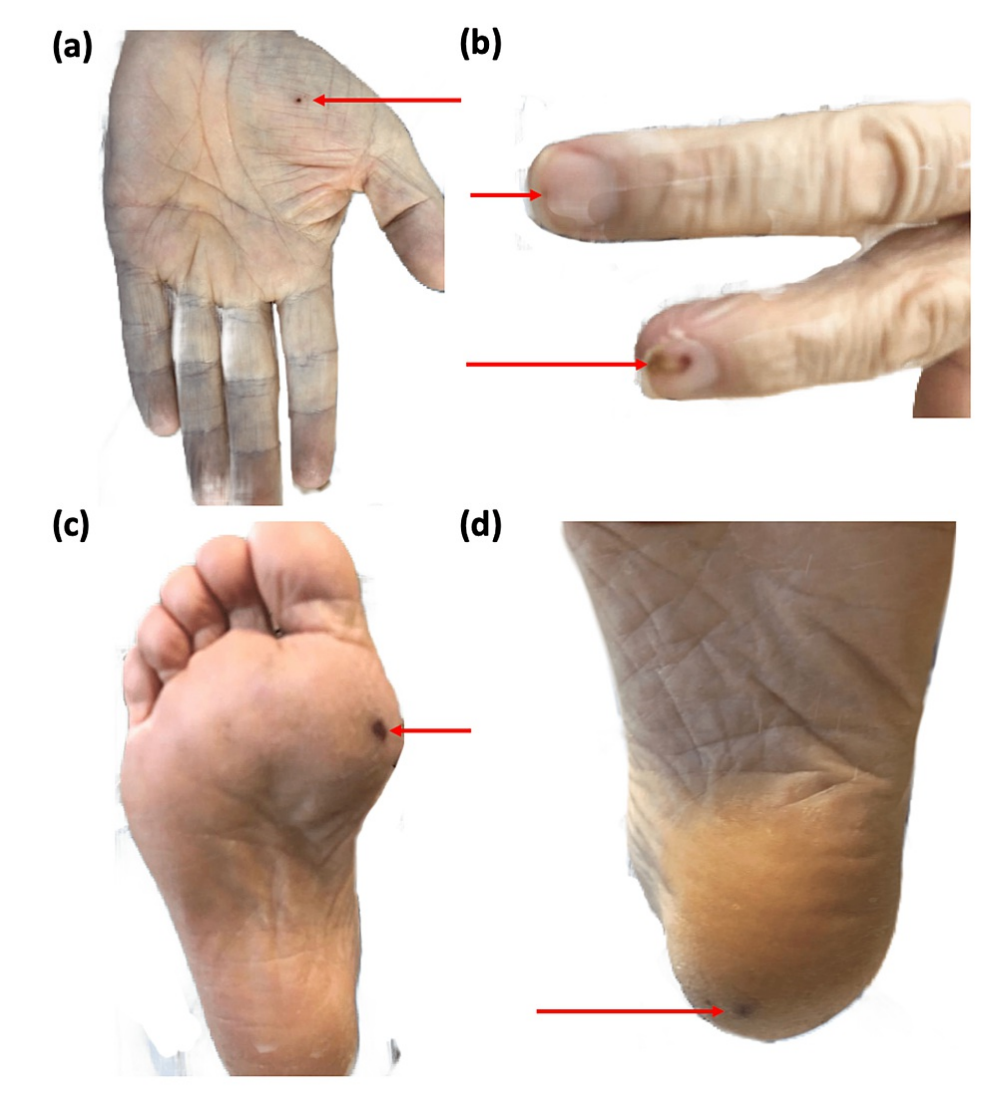
(a) Janeway lesion on the left palm; (b) Splinter haemorrhages on the fingers of the right hand; (c) Janeway lesion on the sole of the right foot; and (d) Janeway lesion on the heel of the left foot.

**Figure 7:**
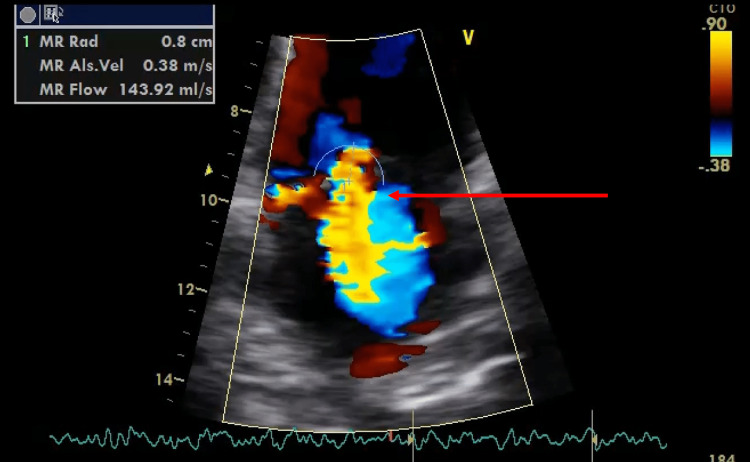
Transthoracic echocardiogram with colour Doppler showing mitral regurgitation.

The patient was transferred to the general ward after eight days in ICU. However, pyrexia persisted despite antibiotic therapy. A pan-systolic murmur was noted over the mitral area with fingernail splinter haemorrhages and Janeway lesions in the palmar and plantar surfaces (Figure 6). Further complications included acute occlusive thrombi in the right basilic and cephalic veins. A repeat TTE demonstrated moderate mitral regurgitation (MR) (Figure 7).

**Figure 8:**
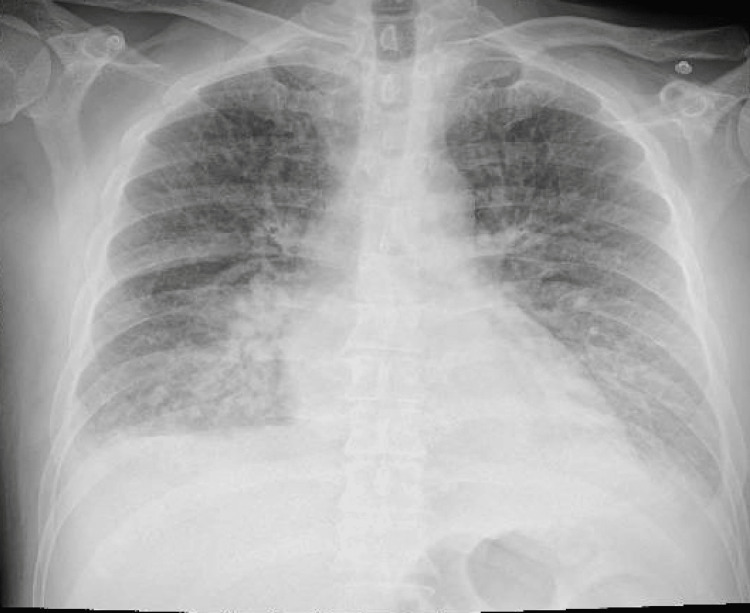
Pulmonary oedema on the anterior-posterior view of the chest X-ray.

The patient subsequently developed acute dyspnoea on minimal exertion associated with oxygen desaturation and bilateral basal crepitations in the lung fields with a heart rate of 110 bpm and a blood pressure of 105/61 mmHg. A chest X-ray confirmed acute pulmonary oedema (Figure 8), which was treated with IV furosemide.

**Figure 9:**
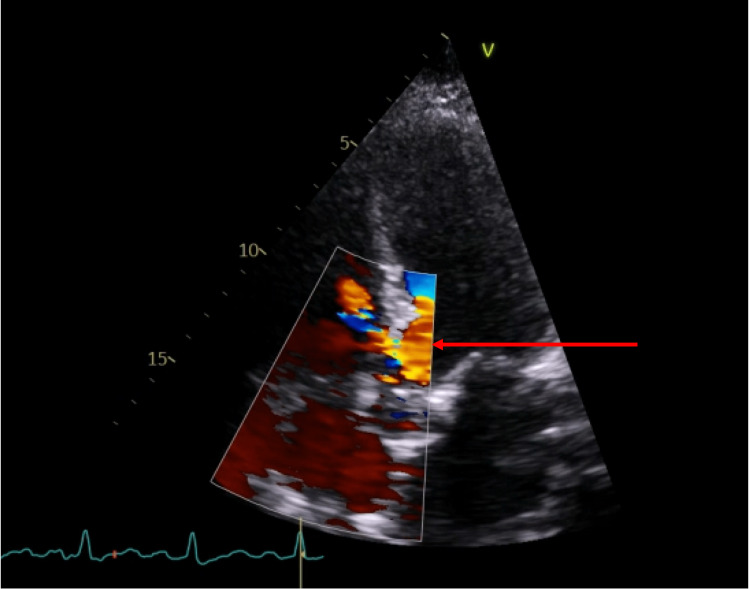
Apical five-chamber view of a transthoracic echocardiogram with colour Doppler indicating flow across the acquired ventricular septal defect.

An urgent repeat TTE was performed, which detected a left-to-right shunt, suggesting a peri-membranous outlet VSD (Figure 9), moderate MR and a mildly dilated left ventricle. 

Definitive management and follow-up

He was sent for urgent cardiothoracic surgery, which revealed vegetation on the right coronary cusp (RCC) of the aortic valve (AV) with an abscess around the AV annulus and through the septum, resulting in a VSD and perforated RCC. The mitral valve was not involved. Following this, the patient underwent tissue AV replacement and bovine patch VSD repair, as well as concurrent double coronary artery bypass graft surgery. 

Post-operatively, there was a right atrial infected thrombus, which was managed conservatively with flucloxacillin and rifampicin. Echocardiography four months post-surgery showed well-seated tissue AV, LVEF 55% to 60% and mild-moderate MR. Six months post-surgery, the patient was symptom-free and returned to his functional baseline and employment.

## Discussion

Acquired VSD secondary to IE is rare and there are only a few such cases reported [4,5]. This case is novel in which the patient had persistent clinical signs and symptoms of IE and subsequently developed VSD despite expedient antimicrobial therapy with resultant negative blood culture and absence of vegetation in multiple echocardiography including a TOE.

The decision for cardiothoracic surgery was the result of the development of fistulation, the VSD and acute pulmonary oedema, all of which met the criteria of the European Society of Cardiology (ESC) guidelines (2015) on the management of IE [7]. 

In this case, IE was caused by the *S. aureus* strain that produces PVL, a cytotoxin that causes tissue necrosis commonly in the form of necrotising pneumonia and skin infection [6]. Another uniqueness of PVL-producing *S. aureus* is that it exerts its virulence factors through biofilm formation [8], which may explain the absence of vegetation in multiple echocardiograms here. While other strains of *Staphylococcus* spp. have been reported to cause IE with acquired VSD [5], to the authors’ knowledge, along with a literature review of acquired VSDs in IE, there has not been any reported case associated with PVL-producing *S. aureus*. 

The 2015 ESC guidelines recognise that *S. aureus* causes acute destructive IE and recommend specific antibiotic therapy targeted against each strain of *S. aureus* [7]. Where no vegetation was detected on TTE or TOE, the recommendation is for a repeat TOE within five to seven days [7]. Given the virulence of PVL-producing *S. aureus* and its potential to cause tissue destruction, this novel case may provide grounds for amendments to the 2015 ESC guidelines to consider the addition of anti-toxin antibiotics like linezolid [9] on top of the usual antibiotic therapies in MSSA bacteraemia such as beta-lactam [10], as well as early cardiothoracic surgical interventions if PVL-producing *S. aureus* is detected in IE even in the absence of vegetations on echocardiography and certainly prior to the development of intracardiac fistula or VSD. 

## Conclusions

PVL-producing *S. aureus* IE can cause tissue necrosis, including VSD. Thus, it is imperative to promptly obtain a blood culture in suspected IE to guide targeted antimicrobial therapy. Anti-toxin antibiotics should be considered if toxin-producing bacteria, such as PVL-producing *S. aureus*, are isolated. Furthermore, IE patients should undergo regular clinical assessment for complications, including the duration of the PR interval. Early cardiothoracic surgical interventions should be considered in the context of PVL-producing *S. aureus* IE even in the absence of vegetation on echocardiography.
